# Post-transarterial Chemoembolization Tumor Rupture in a Patient with Autoimmune Hepatitis Cirrhosis and Hepatocellular Carcinoma

**DOI:** 10.7759/cureus.7750

**Published:** 2020-04-20

**Authors:** Khushboo Gala, John Guardiola-Bright, Suzanne McGee

**Affiliations:** 1 Internal Medicine, University of Louisville Hospital, Louisville, USA; 2 Internal Medicine, University of Louisville, Louisville, USA; 3 Emergency Medicine, University of Louisville, Louisville, USA

**Keywords:** tace, autoimmune hepatitis, hcc, tumor rupture, tace complication

## Abstract

Transarterial chemoembolization (TACE) is a generally well-tolerated and safe procedure that is increasingly being used in the management of intermediate-stage hepatocellular carcinoma (HCC). Tumor rupture is a rare major complication of TACE. Predisposing factors for tumor rupture include large tumor size and peripherally located tumors; in cases of HCC in cirrhosis secondary to autoimmune hepatitis (AIH), tumor rupture may occur more frequently because of the phenomenon of peliosis that occurs in AIH leading to higher propensity to rupture. Management of tumor rupture can be surgical or conservative depending on the individual case. We describe the first documented case of tumor rupture post-TACE in a patient with AIH cirrhosis and HCC.

## Introduction

Hepatocellular carcinoma (HCC) is one of the most common malignancies with over half a million new cases diagnosed globally every year, and is considered to be the fourth most common cause of cancer-related death worldwide [1]. It is well known that patients with cirrhosis from any cause have an increased risk of developing HCC (1%-8%/year); however, patients with cirrhosis secondary to autoimmune hepatitis (AIH) have a relatively lower risk of only 10.07 per 1,000 patient-years (PY), when compared to other etiologies, like hepatitis C virus (3.92/100 PY) and non-alcoholic fatty liver disease (1.2/100 PY) [2-5]. TACE is the first-line treatment for patients with intermediate-stage HCC (Barcelona Clinic Liver Cancer class B); it is also used alone or in combination with other treatment modalities for patients with early or advanced disease [6,7]. TACE is a generally well-tolerated and safe procedure, and various studies have documented a very low rate of major complications (0.84%-2.65%) [8,9]. We describe the first documented case of tumor rupture post-TACE in a patient with AIH cirrhosis and HCC.

## Case presentation

A 76-year-old Caucasian female with a past medical history of cirrhosis secondary to AIH, with previous transjugular intrahepatic portosystemic shunt procedure for esophageal variceal bleeding, was found to have a 6 cm mass in the anterior right lobe of liver on a surveillance abdominal ultrasound. This was followed by an MRI liver mass protocol, which showed a 6.4 x 6.4 cm lesion in the center of the right lobe with early arterial enhancement and washout on delayed phase imaging compatible with HCC. Another 2.1 x 1.6 cm lesion was seen more anteriorly in the gallbladder fossa. She subsequently underwent positron emission tomography scan which showed the larger hepatic lesion and no distant metastases. She was referred for a TACE procedure.

**Figure 1:**
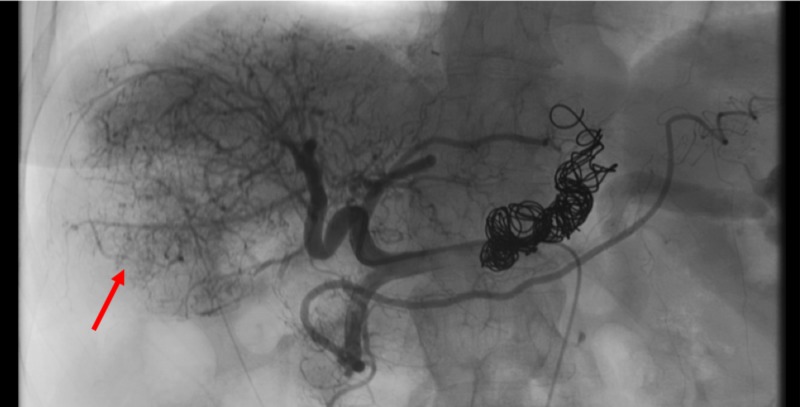
Large hypervascular tumor seen on selective angiogram of the hepatic artery before TACE (red arrow). TACE, transarterial chemoembolization.

The patient underwent TACE with LC beads (Boston Scientific, Marlborough, MA) and doxorubicin (Figure 1).

**Figure 2:**
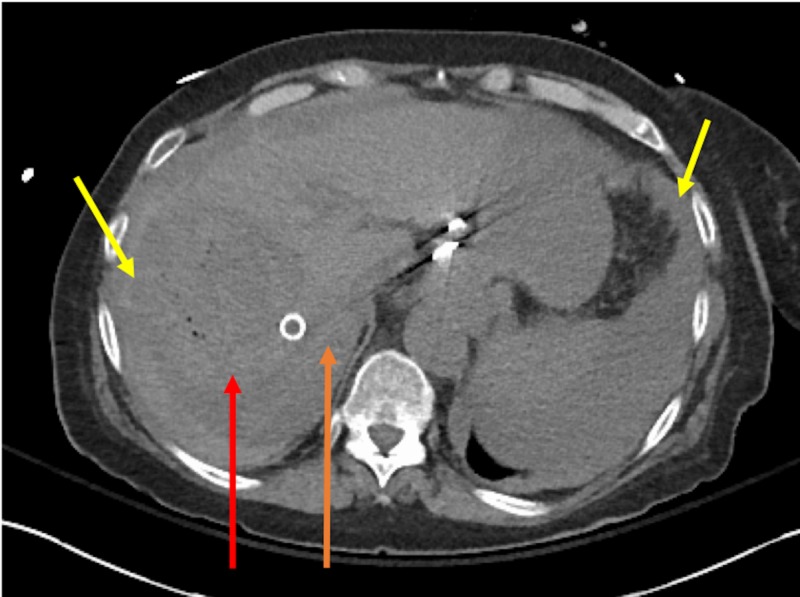
Post-procedure changes seen in tumor showing small foci of gas (red arrow). Loss of wall outline concerning for rupture. Internal hyperdense material concerning for acute bleed. Hemoperitoneum seen near spleen and liver (yellow arrows). Old TIPS seen (orange arrow). TIPS, transjugular intrahepatic portosystemic shunt.

**Figure 3:**
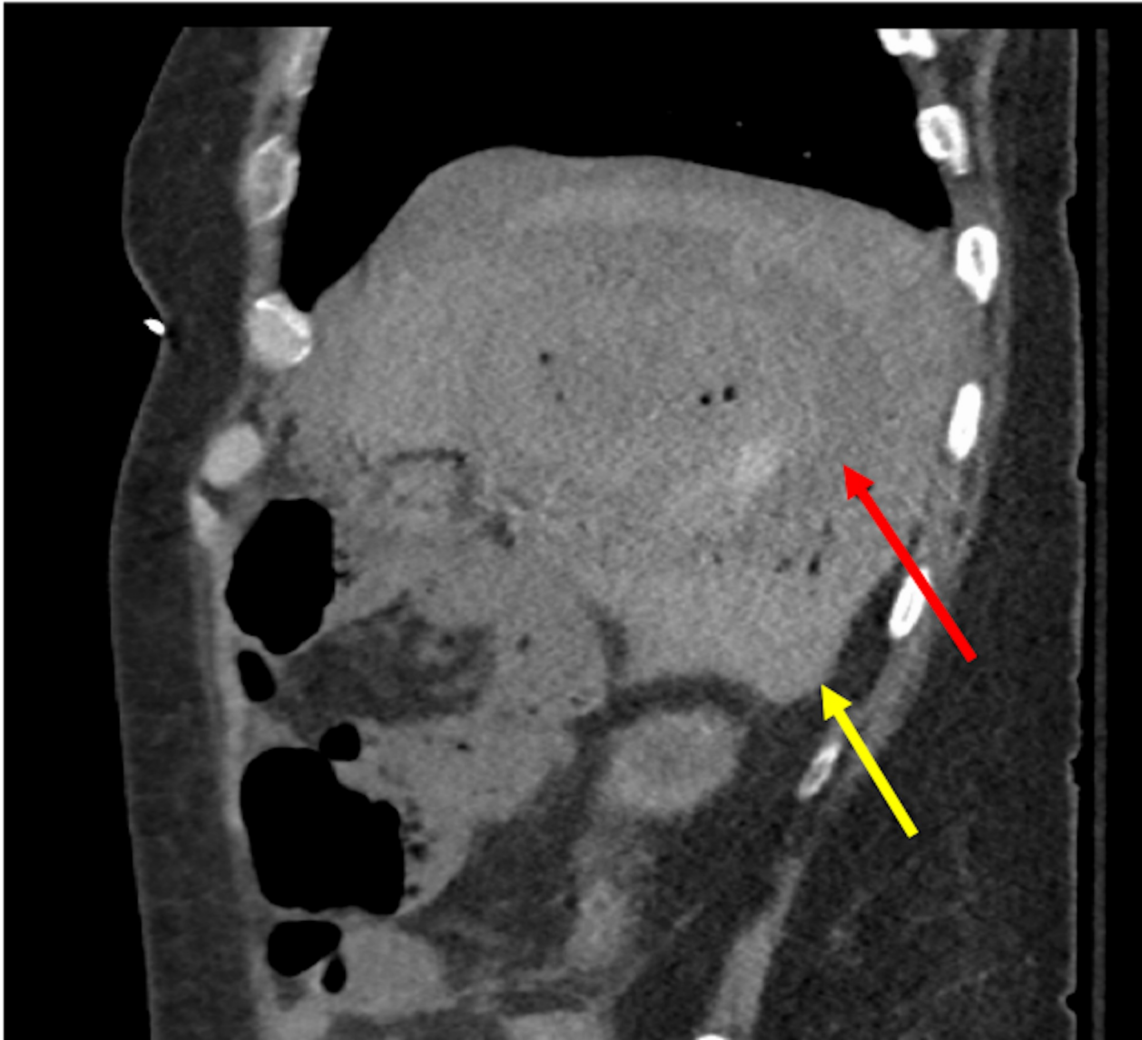
Post-procedure changes seen in tumor showing small foci of gas (red arrow). Sagittal plane: hemoperitoneum seen (yellow arrow).

Due to intractable post-procedure nausea, she was admitted overnight for observation. She did well initially; however, 12 hours post-procedure, she developed acute hypotension requiring vasopressors and was transferred to the intensive care unit. A CT of the abdomen showed moderate volume of hemoperitoneum and free contrast in the upper abdomen worrisome for tumor rupture (Figures 2, 3).

The patient subsequently developed disseminated intravascular coagulopathy, acute kidney injury, and non-ST elevation myocardial infarction secondary to demand ischemia. Trauma surgery and interventional radiology were consulted. Given the patient’s overall condition and multiple comorbidities, the decision was made to manage the patient conservatively. The patient’s condition stabilized with supportive care, and she was able to be moved out of the intensive care unit. Before she could be discharged, she developed hospital-acquired pneumonia leading to respiratory compromise. Given her overall clinical picture of significant acute illness superimposed on serious chronic illness with continued deterioration, the patient and her family elected to change goals of care to comfort measures only and allowing patient to pass away.

## Discussion

TACE involves the injection of a chemotherapeutic agent into the hepatic artery with or without lipiodol and procoagulant material. Complications of TACE are usually self-limited and easy to manage. Approximately 10%-20% of patients suffer from postembolization syndrome, which features fever, right upper quadrant abdominal pain, nausea, and vomiting, and is a benign, self-limiting condition [9,10]. Other minor side effects include mildly impaired liver function, leukopenia, and transient fevers [11]. The Society of Interventional Radiology defines major complications as events leading to an admission to a hospital for therapy, an unplanned increase in the level of care, prolonged hospitalization, permanent adverse sequelae, or death after conventional transarterial embolization/TACE therapy [12]. Major complications described in literature include tumor rupture, liver abscess, femoral artery pseudoaneurysm, pulmonary or cerebral lipiodol embolism, intestinal perforation/obstruction, acute pancreatitis, cholecystitis, and gallbladder perforation [8-10].

Tumor rupture after TACE is extremely uncommon (reported as 0.4%-0.68% per procedure) with mortality reaching as high as 50% [13-15]. A review of literature yields <25 reported cases of the same [15]. Reported predisposing factors are male sex, large tumor size, right lobe and peripherally located tumors, exophytic growth of tumor, first session of TACE, and TACE performed without previous hepatic resection [13,14,16]. Most patients with tumor rupture were reported to have alcoholic, hepatitis B, or hepatitis C cirrhosis. The mean size in tumors that ruptured was >10 cm. Our case is the first documented one of tumor rupture post-TACE in a patient with AIH cirrhosis and HCC.

Spontaneous rupture of HCC has been more widely described in literature, with proposed mechanisms being vascular injury and increased intratumoral pressure [17]. Histopathological analysis of specimens of spontaneous rupture of HCC has shown degradation of type IV collagen, an increase in degenerated elastin and collagenase production, and a significant decrease of von Willebrand factor around the blood vessels [18]. After TACE, injection of chemotherapeutic agents into the tumor causes necrosis of the tumor and tumor wall, leading to increased intratumor pressure and wall fragility, which contribute to risk of rupture. Factors such as large tumor size and peripherally located tumors may also play a role in this process. In AIH cirrhosis, HCC tumor size is actually reported to be smaller than in general HCC cases (in our case, tumor size is 6 cm); however, HCC tumors arising from AIH cirrhosis have been found to have a higher propensity for spontaneous rupture than HCC from other causes [19]. This has been attributed to the peliotic change found commonly in these tumors, which refers to a degenerative change where blood-filled parenchymal cavities are randomly scattered throughout the liver [20]. The peliotic change leads to higher blood flow into the tumor, high intratumoral pressure, and fibrous capsular formation, all proposed to increase the risk of rupture. A high degree of vascular inflammation in cases of AIH may also lead to increased tumor fragility.

Management of tumor rupture depends on a variety of factors. If there is active bleeding and/or peritonitis, patients are frequently managed surgically; in some cases such as ours, treatment is supportive. Given the increasing popularity of TACE, it is important for a practitioner to keep these complications in mind, especially given the associated high mortality. In cases of HCC with AIH cirrhosis, there may be a higher risk of rupture post-TACE, which should be a consideration when selecting therapeutic options and post-procedure monitoring.

## Conclusions

HCC on the background of AIH cirrhosis is very uncommon; however, multiple factors predict that these tumors are more likely to rupture than others. TACE is a therapeutic modality used for the management of intermediate-stage HCC. Tumor rupture post-TACE is a rare but serious complication of an increasingly popular procedure. In patients with HCC and AIH cirrhosis undergoing TACE, it is important to be aware of this complication and monitor them appropriately.
